# Stroke Rate Increases Around the Time of Cancer Diagnosis

**DOI:** 10.3389/fneur.2019.00579

**Published:** 2019-06-07

**Authors:** Yi-Chia Wei, Kuan-Fu Chen, Chia-Lun Wu, Tay-Wey Lee, Chi-Hung Liu, Yu-Chiau Shyu, Ching-Po Lin

**Affiliations:** ^1^Department of Neurology, Chang Gung Memorial Hospital, College of Medicine, Chang Gung University, Keelung, Taiwan; ^2^Institute of Neuroscience, National Yang-Ming University, Taipei, Taiwan; ^3^Community Medicine Research Center, Chang Gung Memorial Hospital, Keelung, Taiwan; ^4^Clinical Informatics and Medical Statistics Research Center, Chung Gung University, Taoyuan, Taiwan; ^5^Department of Emergency, Chang Gung Memorial Hospital, Keelung, Taiwan; ^6^Biostatistical Consultation Center, Chang Gung Memorial Hospital, Keelung, Taiwan; ^7^Department of Neurology, Chang Gung Memorial Hospital, College of Medicine, Chang Gung University, Taoyuan, Taiwan

**Keywords:** stroke, infarction, cerebral hemorrhage, neoplasms, cancer, cumulative incidence, hazard ratio

## Abstract

**Objective:** To test whether strokes increase around the time of cancer diagnosis, we comprehensively examined the correlations of cancer and stroke by employing a population-based cohort study design.

**Methods:** One million people insured under the Taiwan's National Health Insurance program in 2005 were randomly sampled to create the study's dataset. According to the presence of cancer and/or stroke, patients were separated into cancer and stroke, cancer-only, and stroke-only groups. Diagnoses of cancer, stroke, and comorbidities were defined according to ICD9-CM codes. Cancer and non-cancer populations were matched by age at cancer diagnosis, gender, and stroke risk factors, and each patient with cancer was matched with two non-cancer controls nested in the same year of cancer diagnosis. The hazards of stroke and cumulative incidences within a year after cancer diagnosis were evaluated using Fine and Gray's subdistributional hazard model.

**Results:** The temporal distribution of first-ever stroke in patients with both cancer and stroke was a sharpened bell shape that peaked between 0.5 years before and after cancer diagnosis. Frequencies of stroke were further adjusted by number of cancer survivors. The monthly event rate of stroke remained nested around the time of cancer diagnosis in all strokes. Brain malignancies, lung cancer, gastric cancer, prostate cancer, and leukemia patients obtained higher ratio of stroke, while breast cancer and thyroid cancer patients had low percentage of combining stroke. When compared to non-cancer matched control, the hazard of stroke within one year after cancer diagnosis was increased by cancer at a subdistributional hazard ratio of 1.72 (95% confident interval 1.48 to 2.01; *p* < 0.0001).

**Conclusions:** Cancer increased the risk of stroke and stroke events were nested around the time of cancer diagnosis, occurring 0.5 years prior to cancer on average regardless of stroke type.

## Introduction

A study on lung cancer that analyzed data from the National Health Insurance Research Database (NHIRD) found a higher stroke rate just after lung cancer diagnosis (1). Furthermore, the median time from cancer to cryptogenic stroke was 9.6 months in patient data from the cancer registry of Memorial Sloan Kettering Cancer Center and the poststroke survival was less favorable than other patients with stroke with known mechanisms (2). In addition, the Bergen Norwegian Stroke Research Registry found 15.7% patients with stroke had a history of cancer (3). Concealed cancers were evident in several stroke registries (4, 5) with a 5-month mean interval from stroke to the discovery of the concealed cancer (6). These observations suggest a concept in which active cancer and acute stroke codevelop. Relevant studies have either been based on cancer or stroke registries or been confined to a certain type of cancer. Therefore, this study aimed to comprehensively examine the correlations of cancer and stroke by employing a population-based cohort study design.

## Methods

### Database

**Figure 1:**
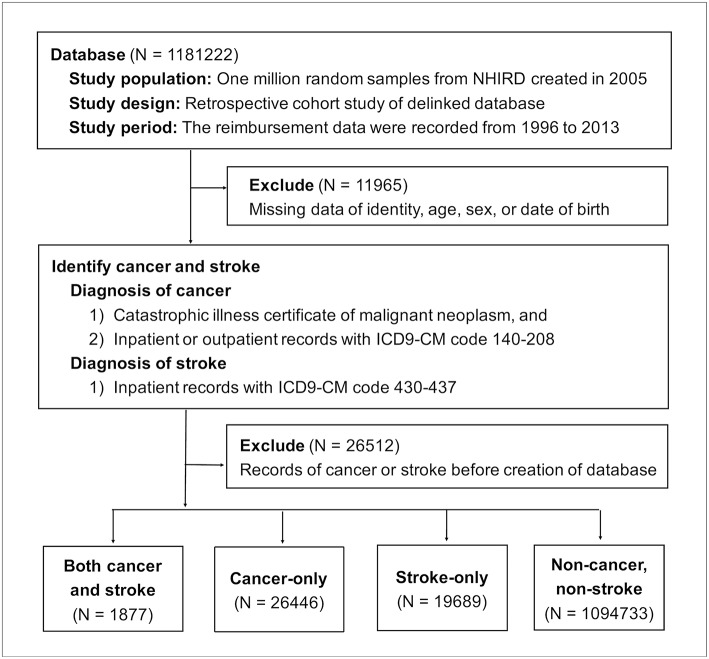
Flow chart of enrollment. NHIRD, National Health Insurance Research Database; ICD9-CM, the Ninth Revision of International Classification of Diseases, Clinical Modification.

Taiwan's National Health Insurance (NHI) program was launched by the Ministry of Health and Welfare in 1995 (7). In this study, 1 million people insured under the NHI program in 2005 were randomly sampled from the NHIRD to create the study dataset. Medical utilization data were traced back to January 1, 1996 and recorded until December 31, 2013. Patients who had either cancer or stroke before January 1, 2005 were excluded. Depending on the presence of cancer and/or stroke, patients were separated into the following groups: cancer and stroke, cancer only, and stroke only (Figure 1).

This study was approved by the Institutional Review Board of Chang Gung Memorial Hospital (No. 201700303B1). Because all the data of NHIRD were de-identified secondary data, individual informed consent was not required. All methods were performed in accordance to the Strengthening the Reporting of Observational Studies in Epidemiology (STROBE) guideline (8). For data availability, anonymized data not published within this article will be made available by request from any qualified investigator.

### Definitions of Cancer, Stroke, and Comorbidities

Diagnoses were based on diagnostic codes of the Ninth Revision of International Classification of Diseases, Clinical Modification (ICD9-CM). Cancer was defined by registration of malignant neoplasm on a catastrophic illness certificate and ICD9-CM code 140-208 (9). In a validation study, NHIRD-based cancer diagnosis had a positive predictive value of 94% when validated by the Taiwan National Cancer Registry (10). Types of cancer were defined by the following ICD9-CM codes: 162 for lung cancer, 153 and 154 for colorectal cancer, 155 for hepatocellular carcinoma, 188 and 189 for urogenital cancer, 151 for gastric cancer, 185 for prostate cancer, 174 for breast cancer, 191 for brain malignancy, 150 for esophageal cancer, 147 for nasopharyngeal cancer, 183 for ovarian cancer, 193 for thyroid cancer, 200–202 and 203.0 for lymphoma, and 204–208 for leukemia.

Stroke events were identified from inpatient payment records using ICD9-CM codes 430–437 (11). Stroke classifications were made according to the validation study with a high degree of accuracy (88.4% positive predictive value and 97.3% sensitivity) (12, 13). ICD9-CM code 430 defined subarachnoid hemorrhage, 431 defined intracerebral hemorrhage, 433 and 434 defined acute ischemic stroke, and 435 defined transient ischemic attack (Table 1). For venous infarction of brain, cerebral venous thrombosis was defined by code 325.0, 437.6, and 671.5 (14).

**Table 1:** Age, sex, and comorbidities of patients with both cancer and stroke, cancer only, and stroke only.

	**ICD9-CM**	**At cancer diagnosis**			**At stroke onset**		
		**Both cancer and stroke**	**Cancer-only**	***p*-value**	**Both cancer and stroke**	**Stroke-only**	***p*-value**
		**(*N* = 1877)**	**(*N* = 26446)**		**(*N* = 1877)**	**(*N* = 19689)**	
Age, year		70.5 ± 13.4	61.1 ± 15.4	<0.0001^*^	70.1 ± 13.2	67.3 ± 14.8	<0.0001^*^
Sex (male)		1203 (64.1 %)	14299 (54.1 %)	<0.0001^*^	1203 (64.1 %)	11249 (57.1 %)	<0.0001^*^
**COMORBIDITY**							
Hypertension	401,405	1531 (81.6 %)	13098 (49.5 %)	<0.0001^*^	1531 (81.6 %)	16895 (85.8 %)	<0.0001^*^
Diabetes mellitus	250	905 (48.2 %)	7849 (29.7 %)	<0.0001^*^	905 (48.2 %)	9655 (49.0 %)	0.496
Chronic kidney disease	585	282 (15.0 %)	2085 (7.9 %)	<0.0001^*^	282 (15.0 %)	2748 (14.0 %)	0.204
Atrial fibrillation	427.31	258 (13.8 %)	959 (3.6 %)	<0.0001^*^	258 (13.8 %)	2803 (14.2 %)	0.580
Coronary artery disease	413, 414.0	634 (33.8 %)	4826 (18.3 %)	<0.0001^*^	634 (33.8 %)	7047 (35.8 %)	0.082
Heart failure	428	441 (23.5 %)	2647 (10.0 %)	<0.0001^*^	441 (23.5 %)	4909 (24.9 %)	0.168
Hyperlipidemia	272	924 (49.2 %)	9273 (35.1 %)	<0.0001^*^	924 (49.2 %)	11055 (56.2 %)	<0.0001^*^
COPD	491, 492, 496	815 (43.42%)	7049 (26.65%)	<0.0001^*^	815 (43.42%)	7408 (37.63%)	<0.0001^*^
Alcohol overuse	V113, 305.0,303, 291, 357.5, 425.5, 571.0, 571.1, 571.2, 571.3, 980.0	78 (4.16%)	1070 (4.05%)	0.816	78(4.16%)	667(3.39%)	0.082
**STROKE CLASSIFICATION**							
SAH	430	—	—	—	30 (1.6 %)	723 (3.7 %)	<0.0001^*^
ICH	431	—	—	—	286 (15.2 %)	3208 (16.3 %)	0.235
AIS	433, 434	—	—	—	1179 (62.8 %)	11975 (60.8 %)	0.091
TIA	435	—	—	—	274 (14.6 %)	2955 (15.0 %)	0.659

* *p < 0.05 was statistically significant. COPD, chronic obstructive pulmonary disease; SAH, subarachnoid hemorrhage; ICH, intracerebral hemorrhage; AIS, acute ischemic stroke; TIA, transient ischemic attack*.

Comorbidities were defined by at least two outpatient records or one inpatient record of the definitive ICD9-CM codes (15). The ICD9-CM codes used to define comorbidities were 401 and 405 for hypertension (11), 250 for diabetes mellitus (11), 585 for chronic kidney disease, (16) 427.31 for atrial fibrillation, (11) 413 and 414.0 for coronary artery disease (11), 428 for heart failure (11) and 272 for hyperlipidemia (11). Alcohol consumption and smoking habit could not be reflected directly by ICD9-CM codes. To overcome this limitation, the consequent diseases represented exposures of alcohol and tobacco (17). Chronic obstructive pulmonary disease (COPD) by ICD9-CM code 491, 492, and 496 represented personal history of tobacco exposure (18). ICD9-CM codes for alcohol-use disorder (V113, 305.0, and 303) and alcoholic organ damages (291, 357.5, 425.5, 571.0, 571.1, 571.2, 571.3, and 980.0) together represented alcohol overuse (19).

### Temporal Correlation of Cancer and Stroke Development

Among the patients with both cancer and stroke, the cancer-to-stroke interval was defined as the time from the date that cancer was first recorded from either inpatient, outpatient, or catastrophic illness certificate records to the date of the first inpatient record of stroke. The zero point of timescales was set as the date that cancer was first recorded. When stroke occurred before the diagnosis of cancer, the cancer-to-stroke interval had a negative value. Only the first stroke event was considered; recurrent strokes were not repetitively recorded.

### Statistics

Cancer and non-cancer populations were matched by age at cancer diagnosis, sex, and stroke risk factors including diabetes mellitus (20), hypertension (20), chronic kidney disease (21), and atrial fibrillation (22). Each patient with cancer was matched with two non-cancer controls nested in the same year of cancer diagnosis. We further tested the competing risk of stroke between cancer patients and non-cancer matched controls. The hazards of stroke and cumulative incidences within a year after cancer diagnosis were evaluated using Fine and Gray's subdistributional hazard model, which considers all-cause mortality as a competing risk (23, 24). All-cause mortality was defined by either (1) remark of death in the catastrophic illness database, (2) either death or critical discharge against medical advice as the status of discharge from hospital AND no new record within 28 days after discharge, (3) discharge against medical advice as the status of discharge AND withdrawal of insurance within 28 days after discharge, or (4) record of resuscitation diagnosis and resuscitation medications in emergency department AND withdrawal of insurance within 28 days (25). The reasons of making the definition of mortality were that NHI is the solitary public health insurance in Taiwan and therefore withdrawals from NHI were rare. Certain people favor to bring their dying families back to home instead of being announced death in hospital. Therefore, the combination of withdrawing insurance and critical discharge was considered mortality. Covariates in the subdistributional hazard model were age, sex, hypertension, diabetes mellitus, chronic kidney disease, atrial fibrillation, coronary artery disease, heart failure, hyperlipidemia, chronic obstructive pulmonary disease, and alcohol overuse. The cumulative event rate of stroke was calculated by dividing cumulative strokes by number of survivors. Statistical analysis was performed using the SAS Enterprise Guide. For continuous variables, independent *t* tests were used to test for statistical significance. For binary data, the chi-squared test was used. A *p* < 0.05 was considered statistically significant.

## Results

### Enrollment and Group Characteristics

Of 1181,222 random samples, 11,965 patients were excluded because of information missing for identity, age, sex, or date of birth. Another 26,512 patients were removed because they had records of either cancer or stroke before the creation of the database on January 1, 2005. Of the 1142,745 enrolled patients during the follow-up period from January 1, 2005 to December 31, 2013, 1,877 patients developed both cancer and stroke, 26,446 patients solely had cancer, and 19,689 patients only suffered stroke (Figure 1).

When we compared the age at time of cancer diagnosis, the cancer and stroke group was older than the cancer-only group (70.5 ± 13.4, 61.1 ± 15.4 years, *p* < 0.0001) and comprised more men (64.1, 54.1%, *p* < 0.0001) as well as more stroke risk factors (hypertension: 81.6, 49.5%, *p* < 0.0001; diabetes mellitus: 48.2, 29.7%, *p* < 0.0001; chronic kidney disease: 15.0, 7.9%, *p* < 0.0001; atrial fibrillation: 13.8, 3.6%, *p* < 0.0001; coronary artery disease 33.8, 18.3%, *p* < 0.0001; heart failure: 23.5, 10.0%, *p* < 0.0001; and hyperlipidemia: 49.2, 35.1%, *p* < 0.0001). When we compared the age at first occurrence of stroke, the cancer and stroke group was older (70.1 ± 13.2, 67.3 ± 14.8 years, *p* < 0.0001) and comprised more men (64.1, 57.1%, *p* < 0.0001) but exhibited less hypertension (81.6, 85.8%, *p* < 0.0001) and hyperlipidemia (49.2, 56.2%, *p* < 0.0001) than the stroke-only group (Table 1). Stroke classifications between groups were not significantly different, except for less subarachnoid hemorrhage (1.6, 3.7%, *p* < 0.0001) in the cancer and stroke group (Table 1).

### Strong Temporal Correlations of Cancer and Stroke Co-development

**Figure 2:**
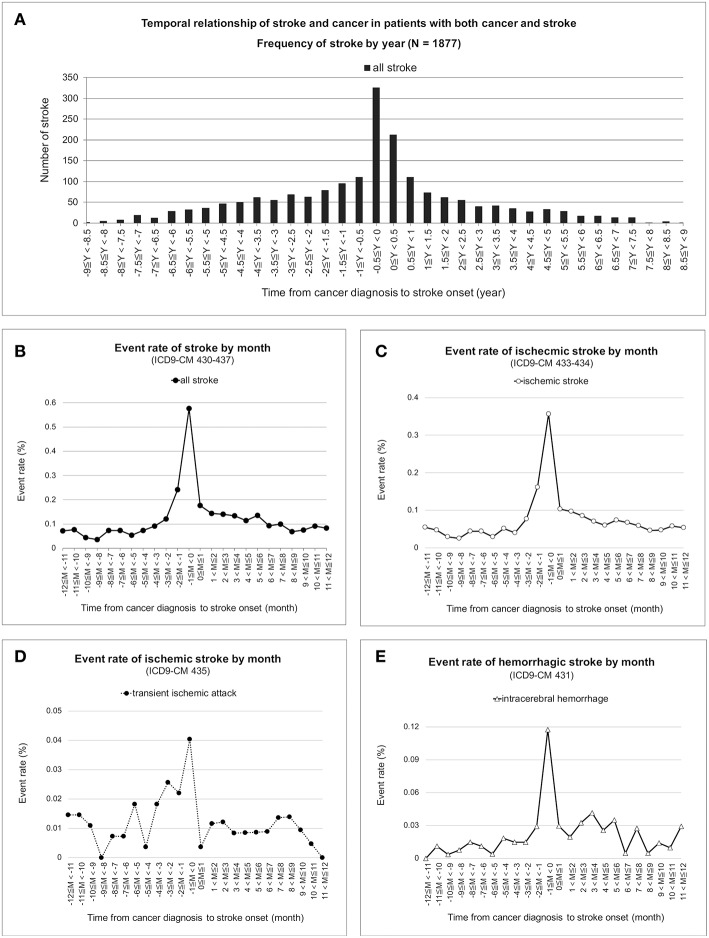
Temporal correlation of cancer and stroke. Frequencies of first stroke nested between 0.5 years before and after cancer was first recorded in the database and were nested around the time of cancer diagnosis in the patients with both cancer and stroke **(A)**. Under time correction of the number of cancer survivors, the monthly event rate of first stroke for all strokes **(B)**, acute ischemic stroke **(C)**, transient ischemic attack **(D)**, and intracerebral hemorrhage **(E)** remained nested and peaked at the time of cancer diagnosis.

The temporal distribution of first stroke in the patients with both cancer and stroke was a sharpened bell shape, which peaked between 0.5 years before and after cancer diagnosis (Figure 2A). Frequency of stroke was further adjusted by the number of cancer survivors. The monthly event rate of stroke remained nested around the time of cancer diagnosis in all strokes (Figure 2B), acute ischemic stroke (Figure 2C), transient ischemic stroke (Figure 2D), and intracerebral hemorrhage (Figure 2E).

### Certain Cancer Types Are Prone to Co-developing Stroke

**Figure 3:**
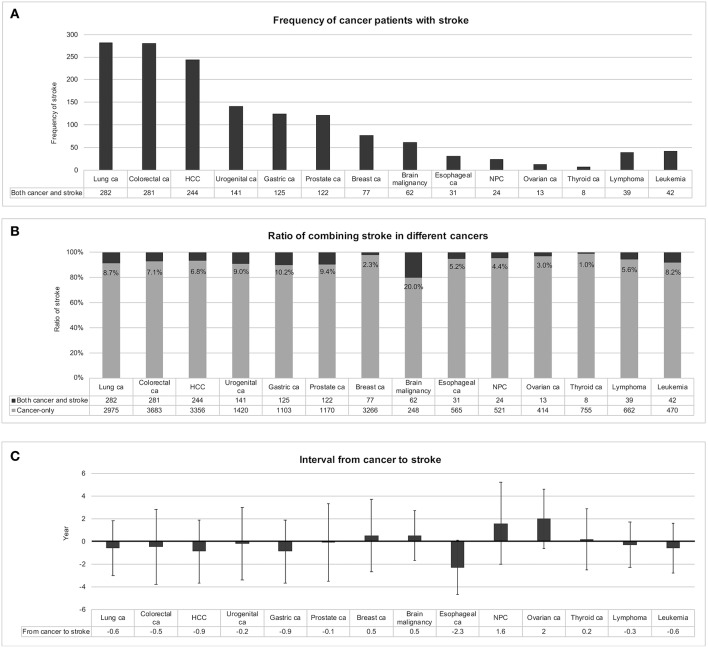
Stroke in different types of cancer. Frequencies of patients with both cancer and stroke were ranked in **(A)**. When patients with stroke were encountered with suspected coexisting cancer, lung cancer, colorectal cancer, and hepatocellular carcinoma should be considered first because of their relatively high frequencies. In individual cancers, certain cancers presented with a relatively high ratio of codeveloping stroke, such as malignant brain tumor, gastric cancer, and prostate cancer **(B)**. The intervals between developing cancer and stroke were also different among cancers **(C)**. Preceding strokes in esophageal cancer and following strokes in nasopharyngeal cancer and ovarian cancer should be considered; otherwise most strokes developed within ± 0.5 years of cancer detection, with a mean interval of −0.4 ± 3.0 years. Abbreviations: ca, cancer; HCC, hepatocellular carcinoma; NPC, nasopharyngeal cancer.

Subsequently, this study ranked the frequencies of various cancers among the 1,877 patients with both cancer and stroke and found that there were 282 (15.0%) lung cancer cases, 281 (15.0%) colorectal cancer cases, 244 (13.0%) hepatocellular carcinoma cases, 141 (7.5%) urogenital cancer cases, 125 (6.7%) gastric cancer cases, and 122 (6.5%) prostate cancer cases (Figure 3A). For each cancer type, the chance of experiencing stroke was ranked from 20.2% for malignant brain tumor, 10.2% for gastric cancer, 9.4% for prostate cancer, 9.0% for urogenital cancer, 8.7% for lung cancer, and 8.2% for leukemia (Figure 3B). The cancer-to-stroke interval was −0.4 ± 3.0 years on average. Some patients developed stroke before cancer, including cancer-to-stroke intervals of −2.3 ± 2.4 years in esophageal cancer, −0.9 ± 2.8 years in hepatocellular carcinoma, and −0.9 ± 2.8 years in gastric cancer. Some other patients experienced stroke after cancer, including stroke onsets of 2.0 ± 2.6 years after ovarian cancer and 1.6 ± 3.6 years after nasopharyngeal cancer (Figure 3C).

### Cancer Increased the Risk of Stroke When Compared With Noncancer Matched Controls

**Figure 4:**
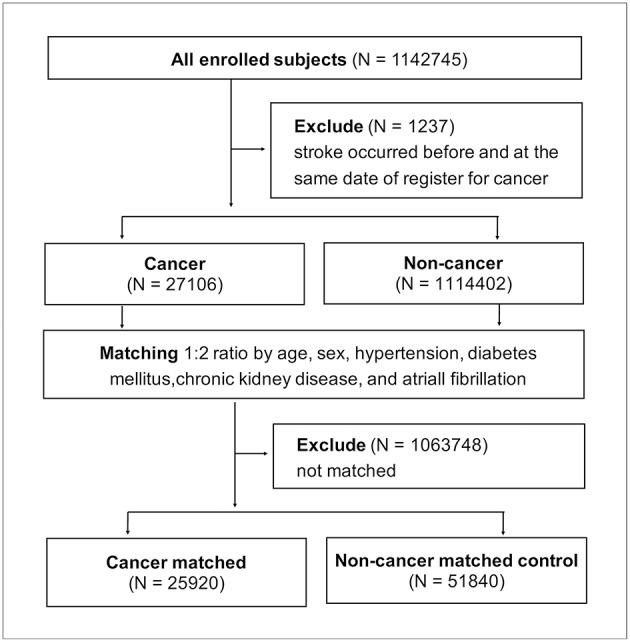
Matching method between cancer and noncancer populations.

All 1142,745 enrolled patients were divided into groups with cancer and without cancer after 1,237 patients were excluded for having stroke records prior to cancer being recorded. The 27,106 patients with cancer were matched at cancer diagnosis to the 1114,402 non-cancer patients at a 1:2 ratio according to the following matching criteria: age, sex, and the presence of hypertension, diabetes mellitus, chronic kidney disease, and atrial fibrillation (Figure 4). After successful matching, 25,920 patients with cancer and 51,840 non-cancer matched controls were entered into a proportional hazards model.

**Figure 5:**
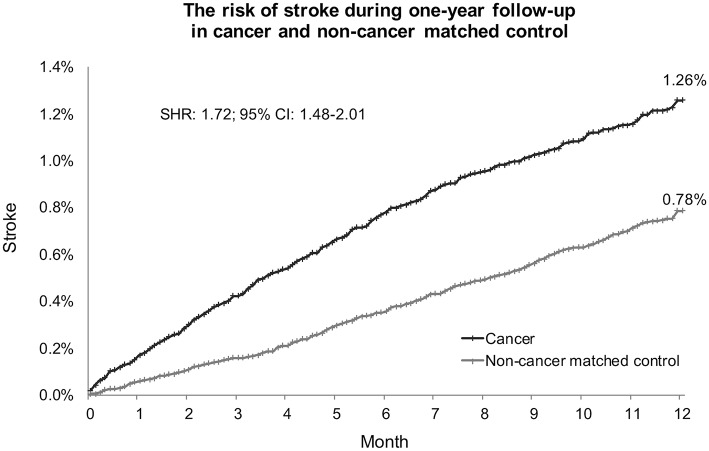
Cumulative stroke event rate in cancer and noncancer matched controls. Subdistribution hazard ratio of stroke increased 1.72-fold in patients with cancer compared with noncancer matched controls under adjustment by all-cause mortality as a competing risk. At the end of the 1-year follow-up, the cumulative rates of stroke were 1.26% in patients with cancer and 0.78% in noncancer matched controls. SHR, subdistribution hazard ratio; CI, confidence interval.

In Fine and Gray's subdistributional hazards model, all-cause mortality was considered a competing risk, the hazard of stroke within a year after cancer diagnosis was increased by cancer at a subdistributional hazard ratio (SHR) of 1.72 (95% confidence interval [CI] 1.48–2.01; *p* < 0.0001; Table 2). The cumulative stroke rates in patients with cancer were higher than those in the non-cancer matched controls; they initially matched and then increased to reach 1.26 vs. 0.78% at the twelfth month of follow-up, respectively (Figure 5). When the comparison period was extended to the end of the cohort follow-up on December 31, 2013, statistics failed to show significant differences in stroke hazards between patients with cancer and non-cancer matched controls (data not shown).

**Table 2:** Competing risk of stroke in patients with cancer and noncancer matched controls.

**Variables**	**Cancer**	**Non-cancer matched control**	**Subdistribution hazard ratio**	**95 % CI**	***p*-value**
	**(*N* = 25920)**	**(*N* = 51840)**			
Cancer	—	—	1.72	1.48 to 2.01	<0.0001^†^
Age, year^*^	60.6 ± 15.2	60.6 ± 15.2	1.04	1.03 to 1.05	<0.0001^†^
Sex (male)^*^	13939 (53.8%)	27878 (53.8%)	1.22	1.04 to 1.43	0.013^†^
Hypertension^*^	11135 (43.0%)	22270 (43.0%)	1.95	1.60 to 2.38	<0.0001^†^
Diabetes mellitus^*^	6188 (23.9%)	12376 (23.9%)	1.41	1.19 to 1.67	<0.0001^†^
Chronic kidney disease^*^	625 (2.4%)	1250 (2.4%)	0.98	0.68 to 1.41	0.900
Atrial fibrillation^*^	211 (0.8%)	422 (0.8%)	2.13	1.42 to 3.20	<0.001^†^
Coronary artery disease	3817 (14.7%)	7211 (13.9%)	1.11	0.92 to 1.34	0.260
Heart failure	1689 (6.5%)	2748 (5.3%)	1.49	1.19 to 1.86	<0.001^†^
Hyperlipidemia	7688 (29.7%)	15061 (29.1%)	0.97	0.82 to 1.15	0.749
COPD	5637 (21.8%)	8591 (16.6%)	1.02	0.86 to 1.21	0.834
Alcohol overuse	905 (3.5%)	687 (1.3%)	1.76	1.16 to 2.66	0.008^†^

*In the subdistributional proportional hazards model, all-cause mortality was considered a competing risk*.

* *These comorbidities were matching criteria of patients with cancer and non-cancer matched controls*.

† *p value < 0.05 was statistically significant. CI, confidence interval; COPD, chronic obstructive pulmonary disease*.

The other variables in the model that increased stroke hazards during the 1-year follow-up period were age (SHR 1.04; 95% CI 1.03–1.05), male gender (SHR 1.22; 95% CI 1.04–1.43), hypertension (SHR 1.95; 95% CI 1.60–2.38), diabetes mellitus (SHR 1.41; 95% CI 1.19–1.67), heart failure (SHR 1.49; 95% CI 1.19–1.86), atrial fibrillation (SHR 2.13; 95% CI 1.42–3.20) and alcohol overuse (SHR 1.76; 95% CI 1.16–2.66; Table 2).

## Discussion

This population-based cohort study examined the co-development of cancer and stroke. A nesting phenomenon of first strokes was noticed around the time of cancer diagnosis, regardless of whether strokes were classified as acute ischemic stroke, transient ischemic attack, or intracerebral hemorrhage. Because of the uneven baselines between the cancer and stroke group and the stroke-only group, cancer and non-cancer populations were matched to control the risk factors. Thereafter, the proportional hazard model demonstrated increased stroke hazards with the presence of cancer.

Because this study was conducted on the basis of data from databases, certain limitations and statistical solutions should be mentioned. First, both cancer and stroke are major diseases with high mortality rates. To avoid survivorship bias, it was necessary to exclude patients with a history of cancer or stroke before creating the database. Furthermore, to correct for possible population decay–related selection bias, timely adjustments of survivor numbers should be performed when encountering incidence statistics and survival analysis (26). When performing hazard estimation of patients with cancer, considering mortality as a competing risk was determined to be crucial (24). Second, because of the limitations of the claims database, patients' stroke-related neurological deficit, performance, independence, brain image, and laboratory data could not be extracted from the database. Cancer staging and pathology were not provided by the NHIRD. Substance and environment exposures could not be directly measured. Alcohol consumption was indirectly estimated by alcoholic organ damage and alcohol-use disorder. Smoking was alternatively coded by chronic obstructive pulmonary disease. Possible biases existed in both indirect representations and inter-individual variations to exposures. Lastly, since all-cause mortality was measured by indirect coding, there's still possibilities of misclassification of death.

The trend of co-development of cancer and stroke has been implied in different registries and database studies. Some cancer registries have found increased risk of stroke in patients with cancer (27), and the risk was high at the onset of cancer (28). Another stroke registry found a cancer history was more common in patients with stroke than in a matched general population (3). Several NHIRD studies have indicated the relationship between cancer and stroke in single cancer types (29–32). For example, an NHIRD study on lung cancer found the highest hazard of stroke in the first 3 months of lung cancer, after which the hazard gradually reduced in the following year (1).

According to our results, most strokes occurred slightly earlier than cancer. A possible mediator is paraneoplastic thromboembolism of active cancer. In addition to stroke, active cancer increases the risk of pulmonary embolism during the peri-cancerous period (33). General considerations of cancer-related cerebral arterial thromboembolism have included paraneoplastic hypercoagulopathy (34, 35), chemotherapy (36), hyperviscosity syndrome in hematologic malignancies (37), Trousseau's syndrome of mucin-positive carcinoma (38), radiotherapy vasculopathy of head and neck cancer (39) and tumor emboli of lung cancer (40). In addition to cancer-specific risks, common vascular risk factors including hypertension, hyperlipidemia, diabetes mellitus, coronary artery disease, and atrial fibrillations have been found to be common in patients with cancer and stroke (41, 42). Therefore, concerns about stroke in patients with cancer are not negligible because of their high risks. Nevertheless, the patients with both cancer and stroke in our study exhibited less hypertension and hyperlipidemia but were older and more often male than the patients in the stroke-only group; therefore, the stroke risk factors were slightly different from the general population. Regarding stroke classifications, there were also fewer subarachnoid hemorrhages in patients with both cancer and stroke. Because most subarachnoid hemorrhages are related to aneurysm rupture or traumatic brain injury (43), this might reflect their non-relevance to cancer-related stroke. In sum, the population features of cancer-related stroke were slightly different from noncancer-related stroke.

The other explanation for the nesting phenomenon of cancer and stroke is surveillance effects. Either active cancer or acute stroke brings people to medical systems more frequently than usual, which results in surveillance bias from frequent medical checkups (44). Therefore, alerts for the codevelopment of cancer and stroke in a short period, the arrangement of diagnostic exams, and prompt treatment are warranted.

Certain types of cancer are more frequently found in combination with stroke. Lung cancer with stroke was the most frequently noticed in the cancer registries (27, 45) as well as in our study. This high frequency could come from hypercoagulopathy, tumor emboli, metastasis to the brain, or perhaps because of the high prevalence of lung cancer itself. Lung, liver and colorectal cancers as the most prevalent cancers in Taiwan (46) codeveloped stroke frequently (Figure 3A). After adjusted with total number of cancer survivors, the percentages of combining stroke in lung, liver, and colorectal cancers were no longer high (Figure 3B). Therefore, high frequency of codeveloping stroke was related to high prevalence of certain cancers. However, breast cancer was also a prevalent cancer but with only 2.3% patients having stroke (Figure 3B). According to Taiwan Cancer Registry, majority of breast cancer patients were diagnosed at stage I or II, especially when mammography screening was promoted by public health policy in Taiwan since 2004 (47, 48). Furthermore, survival rate was high in breast cancer with 5-year-survival rate over 80% (49). Lower stage of cancer and good survival rate was less likely to induce systemic change in coagulation or cancer treatment related complications such as stroke.

Gastric cancer is a prothrombotic cancer by expressing tissue factors in tumor and by increasing coagulation-related proteins (thrombin, fibrinogen, fibrinopeptide A and d-dimer) in blood (50). The prothrombotic nature leads to venous thromboembolism in thirteen percent of gastric cancer patients (51) and might also explains the 10.2% high ratio of codeveloping stroke during whole follow-up period and 4.7% within one year before and after cancer diagnosis (Supplementary Figure 1).

The majority of patients with prostate cancer were elderly with the highest mean age among different cancers (77.4 ± 7.5 years old in prostate cancer with stroke; 73.6 ± 8.8 years old in prostate cancer-only patients). Age is an unmodifiable risk factor for both ischemic and hemorrhagic stroke (52). Androgen-deprivation therapy to treat prostate cancer further added on risk of stroke (53). By multiple causative factors, there's a relatively high percentage (9.4%) of prostate cancer patients codeveloping stroke.

Leukemia as a malignancy of blood cell leads to defected fibrinolysis and releases procoagulants to induce disseminated intravascular coagulation (54). In an epidemiologic study of National Inpatient Sample dataset, active acute myeloid leukemia patients had 50-fold increases of concomitant stroke if compared to all admissions (55). In our dataset, 6.3% of leukemia patients had stroke within one year before and after leukemia (Supplementary Figure 1).

Notably, when we studied the percentages of stroke codevelopment in different cancers, an extraordinarily high percentage (20.0%) was noted in malignant brain tumors (Figure 3B). Brain tumors cause vessel compression, postoperative stroke, and radiotherapy vasculopathy-related stroke (56), intracerebral hemorrhage (57), as well as cerebral venous thrombosis (58). Cerebral venous thrombosis is a relatively low incident stroke type. When we searched the database, only 32 cases were identified. Among them only 2 cases had malignancies which were non-brain (one unspecified solid malignancy and one thyroid cancer). Therefore, role of venous thrombosis in brain tumor related stroke was unable to be demonstrated here. When examining peri-cancerous period, strokes occurred in 13.9% of brain tumor patients within one year before and after cancer diagnosis (Supplementary Figure 1). The radiation therapy induced arterial stenosis was less likely to occur within one year after radiation exposure and was not causative for most brain tumor related stroke (59). Of other stroke inducing factors, primary brain tumor had local effects of hypercoagulation by expression tissue factor (60). Post-operative stroke also accounted half of stroke in a case series of primary brain tumor (56). To summarize, the high prevalence of stroke in brain tumors is explained by disease complications and treatment-specific mechanisms.

In some cancers with delayed onset of stroke, cancer treatment–related mechanisms have been considered. For example, nasopharyngeal cancer is a relatively prevalent cancer in Taiwan (57) and the use of radiotherapy plus chemotherapy and radiotherapy alone for treating nasopharyngeal cancer increased the risk of postcancer stroke (29, 39). Treatment-related stroke, especially radiotherapy-induced carotid stenosis in head and neck cancer required a certain period of time to develop, which was reflected in the mean interval from nasopharyngeal cancer to stroke (1.6 ± 3.6 years in our study) and could continue to accumulate even 10 years after cancer treatment (29).

## Conclusion

Cancer increased the risk of stroke. Stroke events were nested around the time of cancer diagnosis and occurred 0.5 years prior to cancer on average, regardless of whether the stroke was acute ischemic stroke, transient ischemic stroke, or intracerebral hemorrhage. Certain cancers codeveloped with stroke relatively frequently (e.g., lung cancer, colorectal cancer, and hepatocellular carcinoma), whereas some other cancers had relatively high percentages of stroke development, such as malignant brain tumor, lung cancer, gastric cancer, leukemia and prostate cancer. Paraneoplastic complications of cancer, cancer treatment-specific mechanisms, and medical surveillance effects were considered to contribute to the nesting phenomenon of strokes around the time of cancer diagnosis.

## Data Availability

The datasets generated for this study are available on request to the corresponding author.

## Author Contributions

YW and KC wrote the main manuscript text. YW, C-PL, CW, C-HL, and YS designed the study. TL run the statistical analysis. KC supervised statistical methods. YW and TL prepared Figures 1–5.

### Conflict of Interest Statement

The authors declare that the research was conducted in the absence of any commercial or financial relationships that could be construed as a potential conflict of interest.

## Supplementary Material

Supplementary Figure 1 Ratio of stroke within one year before and after diagnosis of different cancers.
