# Supplementary figures and images for: Stroke Rate Increases Around the Time of Cancer Diagnosis
Source: Front Neurol. 2019 Jun 7;10:579. doi: 10.3389/fneur.2019.00579 (PMC6566310; doi:10.3389/fneur.2019.00579)

**Ratio of stroke within one year before and after diagnosis of cancer: different cancers**

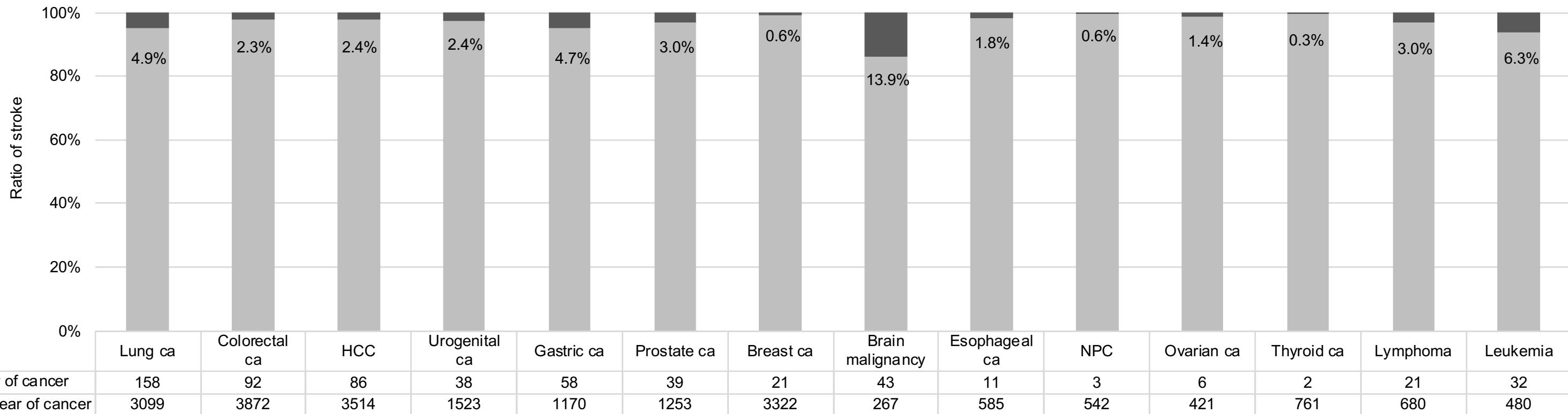

Supplement: Supplementary Figure 1 — Ratio of stroke within one year before and after diagnosis of different cancers. [file Image_1.pdf]
